# *Bartonella henselae* Recombinant Pap31 for the Diagnosis of Canine and Human Bartonelloses

**DOI:** 10.3390/pathogens11020182

**Published:** 2022-01-28

**Authors:** Pradeep Neupane, Ricardo G. Maggi, Manoj Basnet, Erin Lashnits, Gerard P. Andrews, Edward B. Breitschwerdt

**Affiliations:** 1 Department of Clinical Sciences, Comparative Medicine Institute, College of Veterinary Medicine, North Carolina State University, 1060 William Moore Dr., Raleigh, NC 27607, USA; pneupan@ncsu.edu (P.N.); rgmaggi@ncsu.edu (R.G.M.); 2 Department of Electrical and Computer Engineering, University of Memphis, Memphis, TN 38111, USA; mbasnet1@memphis.edu; 3 Department of Medical Sciences, School of Veterinary Medicine, University of Wisconsin, Madison, WI 53713, USA; lashnits@wisc.edu; 4 Department of Veterinary Sciences, College of Agriculture and Natural Resources, University of Wyoming, Laramie, WY 82070, USA; gandrews@uwyo.edu

**Keywords:** antigen, *Bartonella henselae*, serology, diagnosis, ELISA, Western blot, heme binding proteins

## Abstract

*Bartonella* spp. comprise a genus of Gram-negative alphaproteobacteria that are slow growing, fastidious, and facultative intracellular pathogens with zoonotic potential. Immunofluorescent antibody assays (IFAs), Western blot (WB), and enzyme-linked immunosorbent assays (ELISAs), the frequently used modalities for the serological diagnosis of canine and human Bartonelloses, generate numerous false negative results. Therefore, the development of a reliable serodiagnostic assay for Bartonelloses is of clinical and epidemiological importance. Pap31, a heme binding surface protein of *B. henselae,* is associated with bacterial adhesion and related to bacterial colonization. To our knowledge, *B. henselae* Pap31 and its fragments (N-terminal (NTD), middle (MD), and C-terminal (CTD) domains) have not been investigated for the serodiagnosis of canine and human Bartonelloses. In this study, we evaluate the diagnostic utility of *B. henselae* recombinant whole Pap31 (rPap31) and Pap31 fragments by ELISA using sera from 70 dogs (36 *Bartonella* spp. IFA-positive (naturally infected), and 34 *Bartonella* spp. IFA- and PCR-negative (control dogs)) and 36 humans (18 *Bartonella* spp. IFA-positive (naturally infected) and 18 controls)). In the dogs, the area under the curve (AUC) score of recombinant whole Pap31 was 0.714 with a sensitivity of 42% and specificity of 94% at an OD cutoff value of 0.8955. Among the evaluated recombinant Pap31 proteins for the diagnosis of canine Bartonelloses, rPap31-NTD yielded the highest AUC score of 0.792 (95% CI 0.688–0.895) with a sensitivity of 44% and specificity of 100% at a cutoff value of 1.198. In concordance with this finding, rPap31-NTD also had the highest AUC score of 0.747 (95% CI 0.581–0.913) among the Pap31 recombinant proteins for the diagnosis of human Bartonelloses, with 39% sensitivity and 94% specificity at a cutoff value of 1.366. Recombinant whole Pap31 (rPap31) resulted in 72% sensitivity and 61% specificity at a cutoff value of 0.215 for human Bartonelloses. Due to either low sensitivity or questionable specificity, our findings indicate that recombinant Pap31 and the selected fragments may not be appropriate diagnostic targets in detecting anti-*Bartonella* antibodies in *Bartonella*-infected dogs and humans. The findings from this study can be used to further assess the antigenicity and immunogenicity of *B. henselae* Pap31 as a diagnostic target.

## 1. Introduction

*Bartonella* spp. Comprise a genus of Gram-negative alphaproteobacteria that are slow growing, fastidious, and facultative intracellular pathogens with zoonotic potential [1,2,3,4]. *Bartonella* spp. are transmitted by arthropod vectors, including fleas, lice, sand flies, and ticks, by animal bites and scratches, or via direct contact with *Bartonella*-infected clinical specimens [4,5,6]*. Bartonella* spp. are associated with a broad spectrum of clinical signs and pathological abnormalities in dogs [1,2,3,4]. Dogs infected with *Bartonella* spp. develop clinical manifestations that are similar or identical to diseases observed in human patients infected with the same *Bartonella* spp., including bacteremia, encephalitis, endocarditis, fever of an unknown origin, lymphadenomegaly, myocarditis, ocular disease (uveitis), peliosis hepatis, and vasculitis [1,5,6,7,8,9,10]. More than 10 *Bartonella* spp. have been reported to infect dogs, with *B. henselae*, *B. koehlerae*, and *B. vinsonii* subsp. *berkhoffii* being the species or subspecies most frequently identified in sick dogs in North America [1,4]. Despite the recent advances in biomedical sciences, clinical diagnostic approaches to confirm *Bartonella* infection in dogs have not yet been critically investigated.

Currently, the diagnosis of canine and human Bartonelloses is performed by the isolation of *Bartonella* by culture, the amplification of *Bartonella* DNA by PCR, and the detection of *Bartonella* antibodies by serological assays. Although serology can only confirm exposure, immunofluorescent antibody assays (IFAs), Western Blot (WB), and enzyme-linked immunosorbent assays (ELISAs) are most frequently used for the diagnosis of canine and human Bartonelloses [11,12,13,14,15,16,17,18,19,20]. Previously, we reported that the sensitivity of IFA did not substantially improve despite using a panel consisting of eight *Bartonella* IFA antigens, each tested as an independent serological assay [21]. In that study, *Bartonella* antibodies were not detected in 38% of the *Bartonella* spp. bacteremic dogs, which indicated that IFA substantially underestimates the true serological prevalence of *Bartonella* spp. infections in dogs. In concordance with our initial findings of poor IFA sensitivity, using a panel of 3 *Bartonella* IFA antigens, we subsequently confirmed even lower IFA sensitivity (6%) in dogs with hemangiosarcoma, despite the amplification of *Bartonella* spp. DNA from 73% (80/110) of the dogs [22]. Similarly, despite confirming *Bartonella* spp. infection in most (71%) PCR-positive dogs by qPCR and ddPCR amplification, *B. henselae* WB also had a low sensitivity (33%) in dogs with hemangiosarcoma [23]. Due to the low sensitivity of currently available serological assays for the diagnosis of canine Bartonelloses, the development of a reliable serological assay is of clinical diagnostic importance and of substantial epidemiological importance for the analysis of prevalence and risk factor studies.

*Bartonella henselae* Pap31, an outer membrane protein, appears to be an important virulence factor for bacterial attachment and the colonization of mammalian cells, thus promoting the establishment of *B. henselae* infection in the host [24,25,26]. *Bartonella henselae* Pap31, homologous to the hemin binding protein family of *B. quintana,* is also involved in heme acquisition [24,25]. *Bartonella henselae* Pap31 shares 58.4% identity with the heme binding protein A (HbpA) of *B. quintana* and 31.7% identity with OMP31 porin protein of *Brucella melitensis.* Pap31 proteins act as adhesins for fibronectin, heparin, and human umbilical endothelial cells (HUVECs), thereby mediating host–pathogen interactions [24]. The Pap31 protein of *B. bacilliformis*, the etiological agent of Carrion’s disease, has been previously evaluated as a candidate antigen for the development of a reliable serological assay for human Bartonelloses [27]. Another study, characterizing the immunoproteomic profiles of sera collected from cat scratch disease (CSD) and *B. henselae* infective endocarditis patients also identified Pap31 as an immunoreactive candidate protein for the serodiagnosis of Bartonelloses [28].

To our knowledge, *B. henselae* recombinant Pap31 and selected fragments have not been investigated for the serodiagnosis of canine and human Bartonelloses. Therefore, we evaluated the diagnostic utility of *B. henselae* full-length recombinant Pap31 (rPap31) and recombinant Pap31 fragments (N-terminal, middle, and C-terminal domains of recombinant Pap31) by ELISA. Sera from dogs and humans exposed to or infected with *Bartonella* spp. and control groups (presumptively unexposed; *Bartonella* PCR negative and IFA negative) were used to screen recombinant Pap31 proteins and peptides. We hypothesized that *B. henselae* Pap31 protein elicits a sensitive and specific humoral immune response in dogs and humans exposed to *Bartonella* spp. The aims of this study are: (1) to evaluate the sensitivity and specificity of the entire *B. henselae* rPap31 protein; (2) to compare the sensitivity and specificity of the recombinant Pap31 N-terminal domain (rPap31-NTD), middle domain (rPap31-MD), and C-terminal domain (rPap31-CTD); and (3) to evaluate the diagnostic utility of *B. henselae* Pap31 linear B-cell epitopes for the serodiagnoses of canine and human Bartonelloses.

## 2. Results

### 2.1. In Silico Analysis of B. henselae Pap31

**Figure 1:**
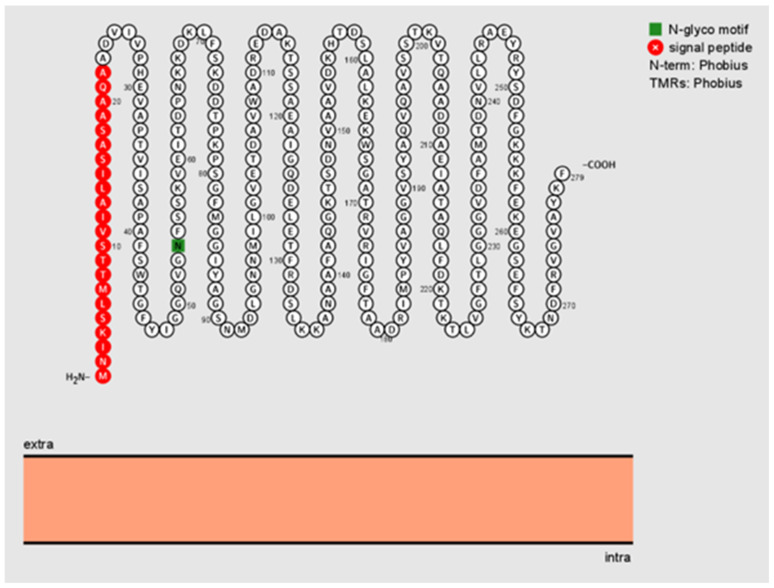
Visualization and predicted sequence features of *B. henselae* Pap31 as determined by Protter, a web-based tool to visualize the sequence, and topology and annotations of individual proteins. The signal peptide sequence is highlighted in red and the potential N-glycosylation site in green. No transmembrane regions (TMRs) are predicted as determined by Phobius. Extra = extracellular; intra = intracellular.

Based on signal peptide analysis, a standard secretory signal peptide (Sec/SPI) was found in *B. henselae* Pap31 with a likelihood probability of 0.9986. Lipoprotein signal peptide (Sec/SPII), Tat signal peptides (Tat/SPI), and other signal peptides were absence in *B. henselae* Pap31 with a likelihood probability of <0.0007. The signal peptide (Sec/SPI) cleavage site was predicted between amino acid positions 22 and 23 with a probability of 0.9902. No transmembrane helices were predicted using TMHMM-2.0, as described in the methods section. *Bartonella henselae* Pap31 protein was predicted to be an outer membrane protein as determined by PSORTb with a localization score of 9.93 and by Protter, Figure 1.

**Figure 2:**
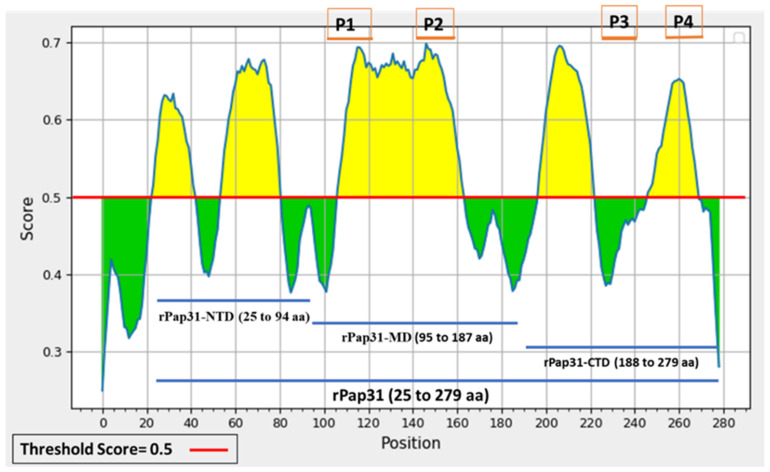
Prediction of linear B-cell epitopes of *B. henselae* Pap31 protein using the IEDB analysis resource webpage BepiPred linear Epitope Prediction 2.0 tool. A threshold score of 0.5 is used for this prediction. Five predicted linear B-cell epitope regions are represented by the area highlighted in yellow. P1, P2, and P4, highly antigenic B-cell epitopes of *B. henselae* Pap31, were selected for testing based on membrane topology and antigenicity scores. P3 was selected based on the prediction from other algorithms described in this study. The regions of Pap31 selected for cloning and purification of recombinant proteins are represented by the blue lines.

In silico analysis of *B. henselae* Pap31 protein using the IEDB Analysis Resource software (BepiPred 2.0) revealed five linear B-cell epitope regions, as represented by the yellow areas above the threshold score of 0.5, Figure 2. The selected regions of the Pap31 protein for the cloning, expression, and purification of rPap31, rPap31-NTD, rPap31-MD, and rPap31-CTD are represented by blue lines in Figure 2. The region ranging from 1 to 24 amino acids of Pap31 was not selected due to the presence of a signal peptide sequence in this region. The location of the selected highly antigenic linear B-cell epitopes of Pap31 (P1, P2, P3, and P4) are represented by yellow boxes, Figure 2. Although the P3 peptide was not predicted as a B-cell epitope by BepiPred 2.0, this region was predicted to be a linear B-cell epitope by the other three algorithms (AAP, ABCPred, and SVMTriP).

### 2.2. Purification of Recombinant Pap31 and Pap31 Domains (N-Terminal, Middle, and C-Terminal Domains)

**Figure 3:**
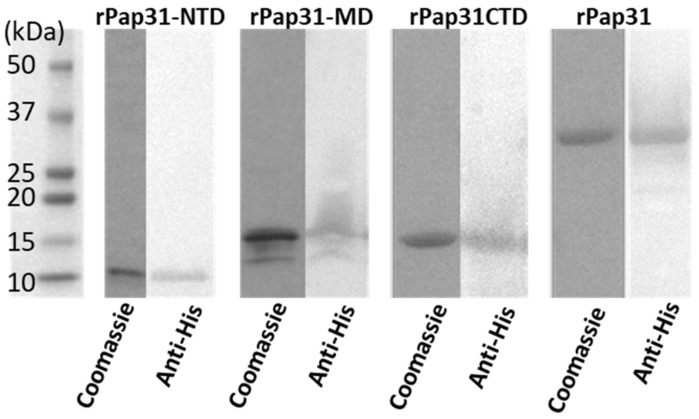
Coomassie stained SDS-PAGE and Western blot analysis of *B. henselae* purified recombinant Pap31-NTD (N-terminal domain), Pap31-MD (middle domain), Pap31-CTD (C-terminal domain), and whole rPap31 (25 to 279 amino acids) proteins. Western blot analysis of purified Pap31 proteins was performed using mouse anti-His antibody (Thermo Scientific, Rockford, IL, USA) and alkaline–phosphatase conjugated Goat anti-mouse IgG (Thermo Scientific, Rockford, IL, USA). (Molecular mass of recombinant proteins Pap31, Pap31-NTD, MD, and CTD were ~31.5 kDa, ~10.5 kDa, ~14.1 kDa, and ~14.1 kDa, respectively. The predicted molecular masses of Pap31 proteins were determined by the coding sequence of the specified Pap31 insert when fused in frame with the pET200D/TOPO expression system fusion tag (~3 kDa)). Coomassie = Coomassie stained SDS-PAGE; anti-His = Western blot analysis.

Sanger sequencing of plasmids isolated from the respective recombinant *E. coli* BL21 (DE3) clones confirmed the insertion of the entire *pap31* gene, as well as the three *pap31* gene fragments into the pET200D/TOPO expression system in the correct reading frame and current orientation. BLAST searches indicated that the in-frame translated amino acid *pap31* gene insert in the recombinant plasmid had 100% homology with *B. henselae* Pap31 (GenBank: AAC39274.1). With the exception of rPap31-MD, purified rPap31 and two rPap31 fragments (rPap31-NTD and rPap31-CTD) yielded a single band, as confirmed by Coomassie stained SDS-PAGE and Western blot analysis, Figure 3.

### 2.3. Seroreactivity of Purified Recombinant Pap31 and Pap31 Domains (N-Terminal, Middle, and C-Terminal Domains) When Tested Using Dog Sera

**Figure 4:**
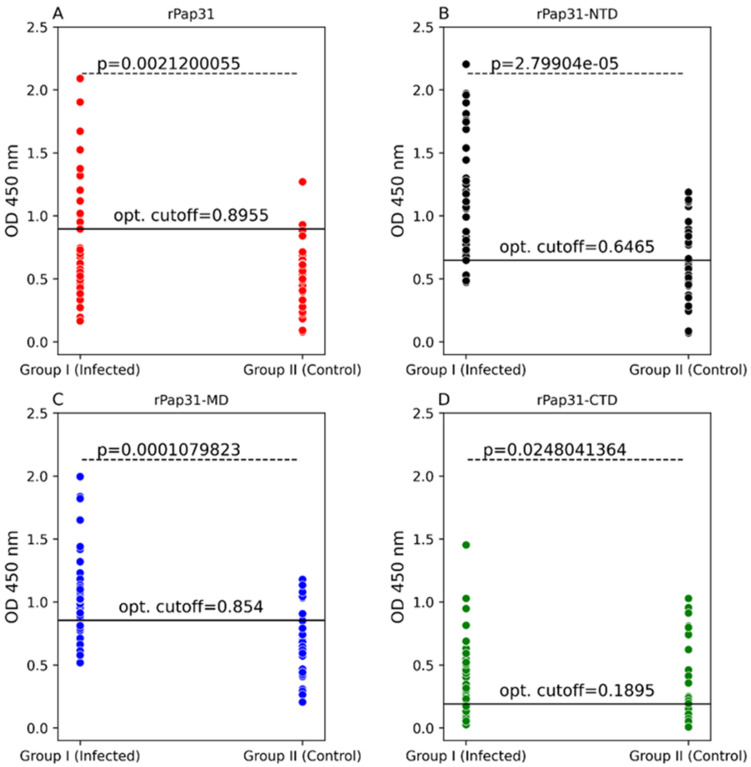
Scatter plots of ELISA seroreactivity among dogs. ELISA seroreactivity for (**A**) recombinant whole Pap31 (rPap31); (**B**) rPap31 N-terminal domain (rPap31-NTD); (**C**) rPap31 middle domain (rPap31-MD); and (**D**) rPap31 C-terminal domain (rPap31-CTD). For ELISA analysis, sera from naturally infected dogs (Group I; n = 36) and control dogs (Group II, n = 34) were used. The difference in ELISA IgG seroreactivity between the two dog groups was determined by the Mann–Whitney U test. The respective *p*-values (dotted line) between the sample groups are given. Optical density cutoff values at a maximum value of the Youden index are represented by the black solid line.

**Figure 5:**
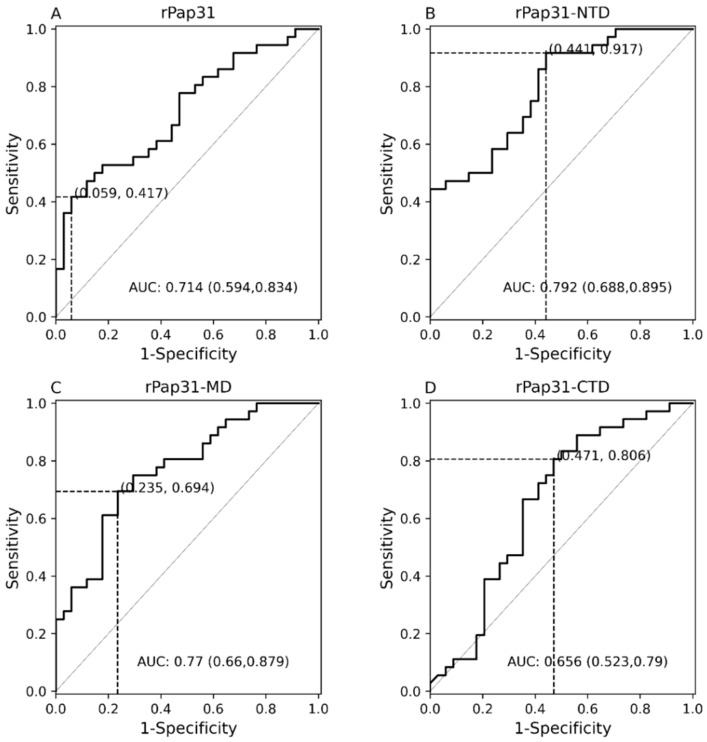
Receiver operating characteristic (ROC) curves of Pap31 ELISA seroreactivity for dogs. The ROC curves for the (**A**) whole recombinant Pap31 (rPap31); (**B**) rPap31 N-terminal domain (rPap31-NTD); (**C**) rPap31 middle domain (rPap31-MD); and (**D**) rPap31 C-terminal domain (rPap31-CTD). The optical density cutoff values were determined to maximize the Youden index. False positive and true positive are shown in parentheses, respectively, at the intersection of the dotted lines. AUC = area under curve.

In this study, the entire length recombinant Pap31 and three domains of rPap31 proteins were tested in an ELISA format using Group I (infected with *Bartonella* spp.; n = 36) and Group II (n = 34 control dogs) for canine Bartonelloses. When testing sera from Group I and II dogs, the sensitivity and specificity of rPap31 was 42% and 94%, respectively, at an OD_450 nm_ cutoff value of 0.8955, with a maximum value of the Youden index as determined by ROC analysis, Figure 4 and Figure 5. An optimal cutoff value was determined using the highest Youden index to maximize sensitivity and specificity. Recombinant Pap31 yielded an AUC of 0.714 (95% CI 0.594–0.834).

Recombinant Pap31-NTD yielded the highest AUC of 0.792 (95% CI 0.688–0.895), Figure 5. At a cutoff value of 0.6465, the rPap31-NTD ROC curve estimated a sensitivity of 92% and specificity of 56% at a maximum value of the Youden index. A higher cutoff value of 1.198 (trade-off between sensitivity and specificity) resulted in 44% sensitivity and 100% specificity for rPap31-NTD. For recombinant Pap31-MD, the sensitivity and specificity was 69% and 76% (at a cutoff value of 0.854), respectively, at the maximum Youden index value, Figure 5. At the higher cutoff value of 1.082, rPap31-MD yielded a sensitivity of 36% and specificity of 94%. We found 81% sensitivity and 53% specificity of rPap31–CTD at the cutoff value of 0.1895 at the maximum Youden index. The AUC score was 0.77 (95% CI 0.66–0.879) and 0.656 (95% CI 0.523, 0.79) for rPap31-MD and rPap31-CTD, respectively. Based on the ROC curve analysis (with an AUC score of > 0.7), rPap31, rPap31-NTD, and rPap31-MD had significant detection (*p* < 0.05) for *Bartonella* antibodies in naturally infected dogs when compared to the controls, Figure 4 and Figure 5.

### 2.4. Seroreactivity of Purified Recombinant Pap31 and Selected Pap31 Domains (N-Terminal, Middle, and C-Terminal Domains) When Tested Using Human Sera

**Figure 6:**
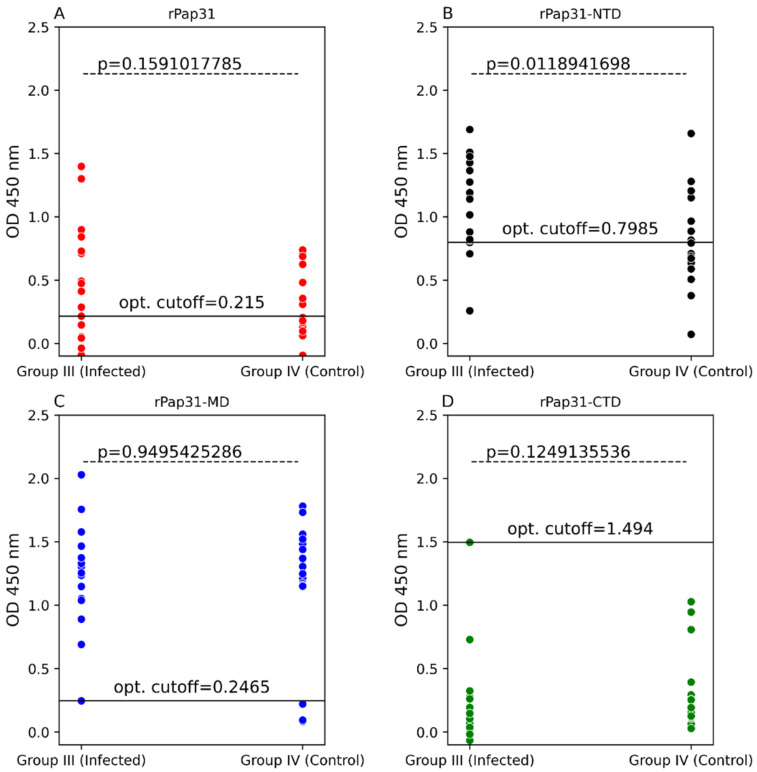
Scatter plots of ELISA reactivity among humans. ELISA seroreactivity for (**A**) recombinant whole Pap31 (rPap31); (**B**) rPap31 N-terminal domain (rPap31-NTD); (**C**) rPap31 middle domain (rPap31-MD); and (**D**) rPap31 C-terminal domain (rPap31-CTD). For ELISA analysis, the sera from naturally infected humans (Group III; n = 18) and control (Group IV, n = 18) were used. The difference in ELISA IgG seroreactivity between the groups was determined by the Mann–Whitney U test. The respective *p*-values (dotted line) between the sample groups are given. The optical density cutoff values at the maximum value of the Youden index are represented by the black solid line.

**Figure 7:**
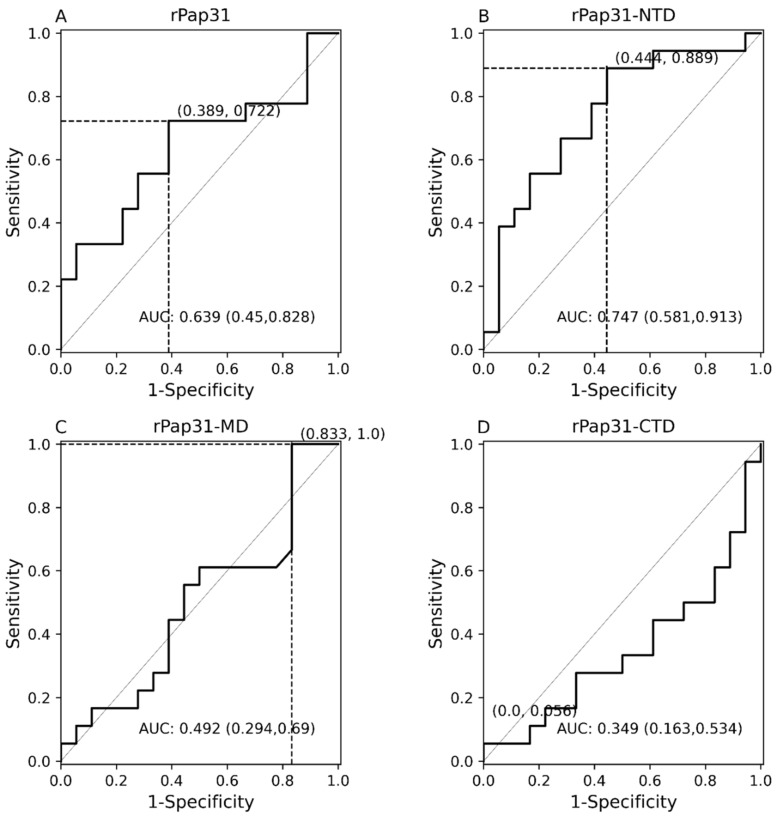
Receiver operating characteristic (ROC) curves with 95% CIs of Pap31 ELISA seroreactivity for humans. The ROC curves for (**A**) whole recombinant Pap31 (rPap31); (**B**) rPap31 N-terminal domain (rPap31-NTD); (**C**) rPap31 middle domain (rPap31-MD); and (**D**) rPap31 C-terminal domain (rPap31-CTD). The optical density cutoff values were determined to maximize the Youden index. False positive and true positive are shown in parentheses, respectively, at the intersection of the dotted line. AUC = area under curve.

Group III (infected with *Bartonella* spp; n = 18) and Group IV (control group; n = 18) human serum samples were tested to evaluate the diagnostic utility of the rPap31 and rPap31 domains for the diagnosis of human Bartonelloses. Recombinant Pap31 resulted in 72% sensitivity and 61% specificity at a cutoff value of 0.215, Figure 6 and Figure 7. The sensitivity and specificity of rPap31-NTD was 89% and 56%, respectively, at a cutoff value of 0.7985, as determined at the maximum Youden index value. A higher cutoff value of 1.366 (trade-off between sensitivity and specificity) for rPap31-NTD resulted in 39% sensitivity and 94% specificity.

There was a significant difference (*p* = 0.01189) in rPap31-NTD reactivity among human Group III compared to Group IV (control group), Figure 6. With the exception of rPap31-NTD, rPap31, rPap31-MD, and rPap31-CTD did not result in the significant detection (*p* > 0.05) of *Bartonella* spp. antibodies when testing naturally infected human sera (Group III) compared to control sera, Figure 6. Based on the ROC analysis, the AUC scores for rPap31 and rPap31–NTD were 0.639 (95% CI 0.45–0.828) and 0.747 (95% CI 0.581–0.913), respectively, Figure 7. The ROC curves for both rPap31-MD and rPap31-CTD yielded AUC scores of <0.5.

### 2.5. Comparison of the ELISA Seroreactivity of Purified Recombinant Pap31 and Pap31 Domains (N-Terminal, Middle, and C-Terminal Domains) among Dogs and Humans

When comparing *Bartonella* spp. IFA- to the rPap31-based ELISAs, the overall percent agreement between *Bartonella* IFA and rPap31-NTD ELISA was the highest (overall percent agreement for dogs and humans was 74% and 72%, respectively), Table 1. Based on the Cohen’s kappa value, there was a moderate agreement between *Bartonella* IFA and rPap31-NTD ELISA (kappa = 0.48) and between *Bartonella* IFA and rPap31-MD ELISA (kappa = 0.46), when the IFA and ELISA assays were performed on the dogs’ sera samples, Table 1. There was fair agreement between *Bartonella* IFA and rPap31 ELISA (kappa = 0.352) and between *Bartonella* IFA- and rPap31-CTD-based ELISA (kappa = 0.337) for the canine Bartonelloses, Table 1. When comparing the *Bartonella* IFA results with ELISA results for human Bartonelloses, there was a fair agreement between *Bartonella* IFA and rPap31 ELISA (kappa = 0.333) and a moderate agreement between *Bartonella* IFA and rPap31-NTD ELISA (kappa = 0.444), Table 1. The agreement between *Bartonella* IFA and rPap31-MD or rPap31-CTD ELISA was slight (kappa = 0.167 and 0.056, respectively) for human Bartonelloses.

**Figure 8:**
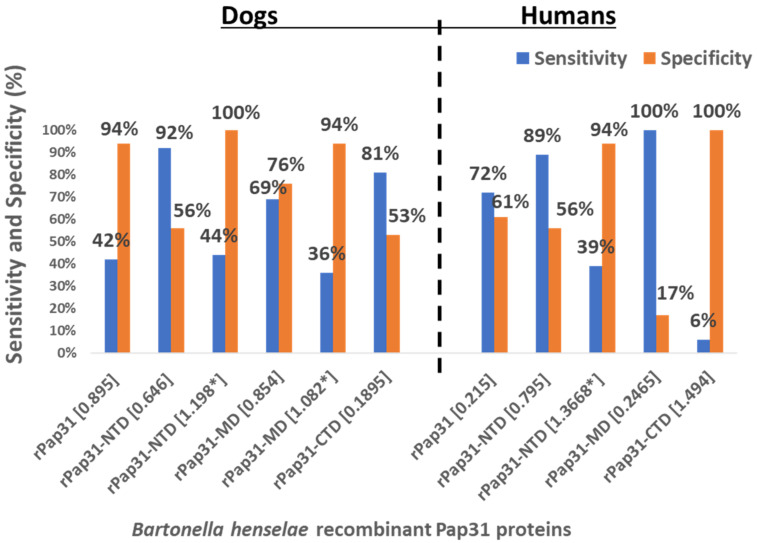
Sensitivity and specificity of *B. henselae* recombinant Pap31 protein-based ELISAs for the diagnosis of canine and human Bartonelloses. Sera from 36 *Bartonella* spp. naturally infected dogs, 34 control (*Bartonella* spp. PCR- and IFA-negative) dogs, 18 *Bartonella* spp. naturally infected humans, and 18 control (*Bartonella* spp. PCR- and IFA-negative) humans were tested by ELISA using whole recombinant Pap31 (rPap31), rPap31 N-terminal domain (rPap31-NTD), rPap31 middle domain (rPap31-MD), and rPap31 C-terminal domain (rPap31-CTD) proteins. The ELISA cutoff OD values for each recombinant protein-based ELISA were determined using ROC curve analysis to maximize the Youden index. * represents higher ELISA cutoff values (trade-off between sensitivities and specificities) for the given recombinant protein-based ELISA.

At an OD cutoff value of 0.646, 33 of the 36 (92%) Group I *Bartonella* IFA-positive dogs were also positive on rPap31-NTD ELISA, while 19 (56%) of the 34 Group II control dogs tested negative by rPap31-NTD ELISA, Table 1. For the human sera, 16 (89%) Group III *Bartonella* IFA-positive and 8 (44%) Group IV controls tested ELISA-positive by rPap31-NTD at a cutoff value of 0.795, Table 1. At higher OD cutoff values, rPap31-NTD resulted in a higher specificity (100% and 94% for dogs and humans, respectively) with low sensitivity values of 44% for dogs and 39% for humans, Figure 8. Among the rPap31 and rPap31 fragments, rPap31-NTD had the highest AUC scores (0.792 and 0.747), respectively, for the diagnosis of both dog and human Bartonelloses, Figure 5 and Figure 7.

### 2.6. Reactivity of Pap31 Linear B-Cell Epitopes Tested with Dog and Human Sera

Despite the coating of ELISA plates with high concentrations of B-cell peptides (P1, P2, P3, and P4) and the use of dog or human sera at 1:50 and 1:100 dilution, none of these peptides were reactive with the sera from *Bartonella henselae* IFA-positive dogs (n = 8) or IFA-positive humans (n = 8) naturally infected with *Bartonella* spp. In addition, using dot blot analysis, these peptides were also not reactive with the same 16 *B. henselae* IFA-positive dog and human sera.

## 3. Discussion

In this study, we evaluated the diagnostic utility of rPap31 and three Pap31 fragments (rPap31-NTD, rPap31-MD, and rPap31-CTD) by ELISA for canine and human Bartonelloses. When testing sera from the dogs and humans that were IFA-positive and/or PCR-positive, the rPap31-NTD and rPap31-MD were the most strongly reactive among the selected recombinant proteins. In dogs, the AUC score of recombinant whole Pap31 (rPap31) was 0.714, with a sensitivity of 42% and specificity of 94% at the OD cutoff value of 0.8955 with a maximum Youden index value, suggesting the diagnostic utility of rPap31 for the diagnosis of *Bartonella* infection in dogs is questionable due to low sensitivity. Among the rPap31 protein fragments, rPap31-NTD yielded the highest AUC score of 0.792 (95% CI 0.688–0.895) with a sensitivity of 44% and specificity of 100% at a cutoff value of 1.198 when testing dog sera, indicating the relatively poor diagnostic sensitivity of rPap31 and the selected fragments for the diagnosis of canine Bartonelloses. In concordance with the findings from dogs, rPap31-NTD also had the highest AUC score of 0.747 (95% CI 0.581–0.913) among the rPap31 protein fragments for the diagnosis of human Bartonelloses, with 39% sensitivity and 94% specificity at a cutoff value of 1.366. Of the rPap31 protein fragments, the ELISA seroreactivity of rPap31-NTD was significantly different when comparing human Group III (naturally infected) and Group IV (control) individuals. However, there was no difference in the reactivity for the two other rPap31 protein fragments when human IFA-reactive sera were compared to control sera. Due to low sensitivity and questionable specificity, our findings indicate that recombinant Pap31 and none of the selected Pap31 fragments are appropriate as diagnostic targets for detecting anti-*Bartonella* antibodies in *Bartonella*-infected dogs or humans.

Nonetheless, rPap31 appears to be highly specific (94%) for the diagnosis of canine Bartonelloses, the specificity of rPap31 was only 61% for the diagnosis of human Bartonelloses. In contrast to the significant differentiation of the infected and control dog groups, there was no significant difference in rPap31 seroreactivity between the infected and control humans. For humans, rPap31 ELISA resulted in 72% sensitivity and 61% specificity, at a cutoff value of 0.215, indicating the potential cross-reactivity of rPap31 with non-*Bartonella* antibodies present in the control group sera. Alternatively, some IFA seronegative humans (control group) may have been exposed to a *Bartonella* spp., but immunofluorescence was not visualized using cell cultured *Bartonella* spp. antigens, as was previously reported (16). In the context of specificity, *B. henselae* Pap31 shares homology with *Neisseria* opacity proteins (Opa), *Brucella* OMP31 (a putative porin), and *Agrobacterium tumefaciens* OMP25 (an immunogenic surface protein) [29,30]. With the exception of rPap31 for the diagnosis of canine Bartonelloses, the rPap31 protein fragments were relatively non-specific at cutoff OD values with a maximum Youden index for the diagnosis of canine Bartonelloses. For human Bartonelloses, rPap31, rPap31-NTD, and rPap31-MD had a specificity of less than 62% at the maximum Youden index value, while the specificity of rPap31-CTD was 100% at the maximum Youden index value. The high degree of identity shared between the *B. henselae* Pap31 protein with proteins of other microorganisms most likely contributed to the low specificity found in this study. These findings indicate that recombinant Pap31 proteins may generate false positive results due to the cross-reactivity with antibodies induced against antigenically similar proteins found in other microorganisms.

A number of factors, such as the selection of cutoff values, antigen preparation, sample population, and standard methods used for the calculation of cutoff values, may influence the assessment of the diagnostic accuracy of an ELISA [31]. The diagnostic accuracy of serological tests has a considerable impact on animal and human health, as well as important economic and epidemiologic implications. In the context of establishing a cutoff value, the sensitivity and specificity of recombinant Pap31 proteins varied at different ELISA cutoff values in this study. At a cutoff value of 0.6465 (with a maximum Youden index), the rPap31-NTD ROC curve estimated a sensitivity of 92% with a specificity of 56% for the diagnosis of canine Bartonelloses. A higher cutoff value of 1.198 (trade-off between sensitivity and specificity) resulted in 44% sensitivity and 100% specificity for rPap31-NTD. For the diagnosis of human Bartonelloses, the sensitivity and specificity of rPap31-NTD were 39% and 94%, respectively, at a higher cutoff value of 1.366 (trade-off between sensitivity and specificity), while rPap31-NTD resulted in 88% sensitivity and a specificity of 56%, respectively, at a cutoff value of 0.7985 with a maximum Youden index. These findings suggest the ELISA cut-off values must be selected with the utmost care, since the selection of a cut-off value becomes the basis for the calculation of sensitivity and specificity, which determines the accuracy of the test result that will be used diagnostically for patient management and disease control strategies.

Logically, *Bartonella* spp. proteins that are associated with survival, multiplication, or bacterial adaptation in accidental and reservoir hosts should be useful targets for the diagnosis of Bartonelloses. Additionally, certain proteins that are important for the pathogen’s survival can be useful for vaccine development but not for serological diagnosis, because of their low antigenicity and high immunogenicity. As a hallmark of these intra-erythrocytic bacteria, the penetration of erythrocytes is an invasion strategy used to obtain heme, which is essential for the growth of *Bartonella* spp. in vivo and in vitro [24,25,26,30,32]. Heme binding proteins (Hbps) of several *Bartonella* spp., including the heme binding protein A (HbpA) of *B. quintana* and Pap31 of *B. henselae*, play an active role in hemin acquisition, survival, and disease pathogenesis [25,26,30]. Therefore, previous studies suggested that heme binding proteins, including Pap31 proteins, are potential candidates for the development of diagnostic and vaccine candidates for Bartonelloses [27,33,34]. Previously, while assessing *B. bacilliformis* recombinant Pap31 for the diagnosis of Oroya fever, the authors demonstrated that Pap31 antigens were highly induced in growing cultures of *B. bacilliformis* and were immunologically recognized dominant proteins in infected humans, supporting the application of Pap31 for the ELISA and WB diagnosis of human Bartonelloses [27]. Dichter et al. (2021), using a reverse vaccinology approach in conjunction with an immunoproteomic approach, also highlighted the potential utility of Pap31 as an immunodominant target recognized by the serum samples obtained from Peruvian patients infected with *B. bacilliformis* [35]. In another study, *B. bacilliformis* recombinant pap31 was not reactive with sera from patients with *Coxiella burnetti, Brucella* spp., or *B. henselae* infection*,* supporting a lack of recombinant *B. bacilliformis* Pap31 cross-reactivity with these microorganisms [34]. In contrast to *B. bacilliformis* findings, *B. henselae* recombinant Pap31 proteins were reactive with sera from a number of dog and human controls in this study. Our findings indicate that the optimization of a recombinant Pap31 protein-based serological assay, perhaps by combining rPap31 fragments in a chimeritope may be needed to enhance sensitivity, specificity, and diagnostic accuracy for the diagnosis of human Bartonelloses.

Despite the predicted high antigenicity scores of the four selected antigenic Pap31 B-cell epitopes (P1, P2, P3, and P4), none of these epitope peptides were reactive with sera from *B. henselae* IFA-positive dogs or humans. Post-inoculation sera from dogs that were experimentally infected with either *B. henselae* San Antonio 2 or *B. henselae* CSU 1 strains, and dogs experimentally infected with *B. vinsonii subsp. berkhoffii* genotype III, were also not reactive to these synthetic peptides, suggesting that three-dimensional epitope conformation is likely of critical importance for the documentation of Pap31 seroreactivity. These findings are consistent with the poor seroreactivity results of *B. bacilliformis* Pap31 linear epitopes reported in a previous study [35], where only 2 of the 26 Peruvian patients with *B. bacilliformis* infection were reactive to *B. bacilliformis* Pap31 linear epitopes in line blots. It is also possible that *B. henselae* Pap31 peptide epitopes represent a minimally immunogenic region of the *B. henselae* Pap31 protein, and hence were not reactive with sera from *B. henselae* IFA-positive dogs and humans in this current study.

The selection of true negative control (*Bartonella* spp. PCR- and IFA-negative) samples are critical for assessing the specificity of serological assays for the diagnosis of Bartonelloses. A limitation of this study is the potential inclusion of serum samples from sick dogs and humans into the respective *Bartonella* spp. PCR- and IFA-negative control groups, which would negatively impact our assessment of ELISA specificity for the diagnosis of canine and human Bartonelloses. Dog sera were submitted to the NCSU-CVM-VBDDL by veterinarians to test for evidence of exposure to or infection with canine vector-borne disease (CVBD) organisms; therefore, despite negative IFA (with a documented poor sensitivity) and PCR results (similarly poor sensitivity in association with chronic infection), these dogs could have been previously exposed to 1 or more of the 40 plus *Bartonella* species [4,21,23]. As an example, despite being obtained from specific pathogen-free dogs maintained in a vector free facility, the dogs infected with *Rickettsia rickettsii* appeared to reactivate occult and previously undetected *Bartonella* spp. infections [36]. Thus, despite negative IFA and PCR testing, our experimental dog control sera may have originated from dogs that had experienced prior *Bartonella* spp. environmental exposures [36,37] and Breitschwerdt EB et al. (unpublished data). Similarly, it is possible that our human controls were naturally exposed to *Bartonella* infection and misdiagnosed as unexposed to or uninfected by *Bartonella* PCR, BAPGM, and IFA testing due to the less than perfect sensitivity of each of these diagnostic assays [19,38,39]. Another limitation of this study is that the blood samples from the 13 Group II control dogs were not processed in BAPGM enrichment blood culture, which is often necessary to confirm *Bartonella* infection in healthy and sick dogs [5,36,40].

Another limitation of this study is the lack of historical and clinical information for the diagnostic specimens submitted for NCU-CVM-VBDDL testing. Although all Group III human patients were tested because of a self-reported history of chronic illness, these sera represented a very heterogenous sample set in the context of symptomatology and duration of illness. This information would be of importance to compare Pap31 seroreactivity versus disease status and duration. Additionally, only *Bartonella* testing results were available (evidence of exposure to phylogenetically-related pathogens, such as *Brucella*, were not available) for human sera. Therefore, the potential cross-reactivity of recombinant Pap31 proteins with sera from humans infected with phylogenetically related pathogens was not addressed in our study. In addition, the information on infecting *Bartonella* spp., genotype, or strain was lacking for 33 Group I (IFA-positive) dogs and 9 Group III (IFA-positive) humans. NCBI Blast searches indicate that *B. henselae* Pap31 shares 50% to 100% identity with Pap31 sequences of other *Bartonella* spp., including medically important animal and human pathogens: *B. henselae* Pap31 (92 to 100%)*, B. koehlerae* Pap31 (83%), *B. quintana* Pap31 (55%), and 50% with *B. bacilliformis* Pap31 (50%). Based upon IFA testing, the sera used in this study may or may not have detected antibodies to all known *Bartonella* spp., genotypes, or strains, which further complicates the evaluation of Pap31-based ELISA sensitivity and specificity.

In conclusion, with additional assessment and optimization, the recombinant Pap31 and rPap31-NTD fragment may be appropriate for canine Bartonelloses diagnostic applications, whereas rPap31-NTD may have application for the diagnosis of human Bartonelloses. The findings from this study can be used to further assess the antigenicity and immunogenicity of *B. henselae* Pap31 as a diagnostic target. Although our results are potentially promising, further studies are needed to optimize a *B. henselae* Pap31-based ELISA for the diagnosis of *Bartonella* infection in dogs and humans.

## 4. Materials and Methods

### 4.1. Serum Samples for ELISA Testing

The *Bartonella* PCR, *Bartonella* droplet digital PCR (ddPCR), and *Bartonella* IFA testing results for the dog and human clinical samples that were used for comparative ELISA in this study are summarized in Table 2 and Supplementary Table S1. IgG titers of ≥1:64 were considered positive for *Bartonella* exposure. In this study, the serum samples from 70 dogs (Group I: 36 *Bartonella* spp. IFA-positive (naturally infected) and Group II: 34 *Bartonella* spp. IFA- and PCR-negative (control dogs)) and 36 humans (Group III: 18 *Bartonella* spp. IFA-positive (naturally infected) and Group IV: 18 *Bartonella* spp. IFA- and PCR-negative (control humans)) were tested by rPap31, rPap31-NTD, rPap31-MD, and rPap31-CTD-based ELISA. All 70 dogs’ sera were submitted to NCSU-VBDDL for diagnostic testing between 2016 and 2019. All 36 humans’ sera were obtained from the repository maintained by the NCSU-CVM-IPRL (NCSU IRB approval# 1960) between 2009 to 2020.

#### 4.1.1. Dog Serum Samples

Seventy archived sera from dogs previously tested at the North Carolina State University, College of Veterinary Medicine, Vector Borne Diseases Diagnostic Laboratory (NCSU-CVM-VBDDL) or the Intracellular Pathogens Research Laboratory, NCSU-CVM (NCSU-CVM-IPRL) were selected for comparative ELISA testing utilizing each of the purified recombinant Pap31 proteins, Table 2 and Supplementary Table S1. The serum samples were categorized into two groups to assess the sensitivity and specificity. All the sera were stored frozen after being submitted to the NCSU-CVM-VBDDL for diagnostic testing between 2016 and 2020. After the initial processing by the NCSU-CVM-VBDDL, sera were stored at −80 °C. Group I consisted of 36 stored frozen serum samples from *Bartonella* spp. naturally infected dogs (*Bartonella* IFA-positive). A cutoff titer of ≥1:64 was used to define an IFA-positive titer. Of the 36 dogs, 1 dog had a *B. henselae* IFA IgG titer of 1:64, whereas the remaining 35 dogs had *B. henselae* IFA IgG titers of ≥1:128. Of these 36 dogs, 23 were concurrently *B. vinsonii* subsp. *berkhoffii* genotype I seropositive (IFA titer range ≥ 1:64 to 1:4096) and 32 dogs were *B. koehlerae* seropositive (IFA titer range ≥ 1:64 to 1:4096). A total of 21 of the 36 dogs were IFA-positive (titers ≥ 1:64) to all 3 *Bartonella* spp. (*B. henselae* San Antonio type *2, B. vinsonii* subsp*. berkhoffii* genotype I*,* and *B. koehlerae*). Of the 36 Group I dogs, *Bartonella* DNA was amplified from the blood of 3 dogs: 2 dogs were PCR-positive for *B. vinsonii* and 1 dog was PCR-positive for *B. henselae,* Table 2.

Based on NCSU-CVM-VBDDL serological and PCR testing, 24 of the 36 Group I dogs were seronegative for evidence of exposure and PCR-negative for evidence of infection with other canine vector-borne disease (CVBD) organisms that are routinely tested for in the NCSU-CVM-VBDDL. Detailed methods for the *Bartonella* PCR, BAPGM enrichment blood culture, and the IFA serological panel used to test these study participants were previously published [14,21]. Specifically, all sera were IFA-negative (titers < 1:16) to *Rickettsia rickettsii*, *Ehrlichia canis*, *Babesia canis*, and *Babesia gibsoni*, and were seronegative to *Anaplasma phagocytophilum*, *Anaplasma platys*, *Borrelia burgdorferi*, *Ehrlichia canis*, and *Ehrlichia ewingii* by ELISA (SNAP 4Dx PLUS ELISA, IDEXX Laboratories, Westbrook, Maine). The blood from these 24 dogs was PCR-negative after whole blood DNA purification for *Babesia*, *Ehrlichia*, *Anaplasma*, *Rickettsia*, hemotropic *Mycoplasma*, and *Leishmania* spp.

Of the remaining 12 dogs, 4 were seropositive as follows: 1 dog was seroractive to *R. rickettsii* (IFA titer 1:128), *Babesia canis* (IFA titer 1:2048), and *Babesia gibsoni* (IFA titer 1:2048); 1 dog was seroractive to *R. rickettsii* (IFA titer 1:512) and *Babesia canis* (IFA titer 1:64); 1 dog was seropositive to *Babesia canis* (IFA titer 1:1024) and *Babesia gibsoni* (IFA titer 1:4096); and 1 dog was seropositive to *Ehrlichia canis* (IFA titer 1:2048). The CVBD PCR and serological testing results were not available for the remaining eight *Bartonella* spp. IFA seropositive dogs.

Group II consisted of 34 dogs for which diagnostic testing in the NCSU-CVM-VBDDL and NCSU-CVM-IRPL did not provide evidence of the exposure to or infection with a *Bartonella* spp., Table 2 and Supplementary Table S1. These sera were used to assess the specificity of the Pap31 recombinant ELISA assays. All of these sera were IFA nonreactive (titers < 1:16) to the three *Bartonella* spp. *(B. henselae* San Antonio type *2, B. vinsonii* subsp*. berkhoffii* genotype I*,* and *B. koehlerae).* All 34 Group II dogs were PCR-negative after whole blood DNA extraction for *Bartonella* spp. Whole blood EDTA from 21 of 34 Group II dogs was processed in *Bartonella* Alpha Proteobacteria Growth Medium (BAPGM) in the NCSU-CVM-IPRL, as described previously [14]. *Bartonella* DNA was not amplified from any extracted blood sample before and after the BAPGM enrichment blood culture. *Bartonella* DNA was also not amplified from reagent controls or BAPGM-negative (un-inoculated) culture controls. Bacterial growth was not visualized following the direct culture of blood or subculture from BAPGM onto Trypticase soy agar II (TSA) supplemented with 5% sheep blood plates. Due to the inadequate blood volumes for BAPGM enrichment blood culture, testing results were not available for the remaining 13 dogs.

Sixteen Group II dogs were PCR-negative after whole blood DNA extraction for *Babesia*, *Ehrlichia*, *Anaplasma*, *Rickettsia*, hemotropic *Mycoplasma*, and *Leishmania* spp., and were seronegative to *Anaplasma phagocytophilum*, *Anaplasma platys*, *Borrelia burgdorferi*, *Ehrlichia canis*, and *Ehrlichia ewingii* by ELISA (SNAP 4Dx PLUS ELISA, IDEXX Laboratories, Westbrook, Maine) or in-house IFA assays. A total of 10 of these 16 dogs were *R. rickettsii* seropositive (IFA titer of ≥1:64). *Rickettsia rickettsii* is phylogenetically related to the genus Bartonella, belonging to the class alphaproteobacteria [41]. Therefore, to further assess specificity, *R. rickettsii* IFA-positive dogs were included in the Group II dogs. Additional serological testing for the exposure to CVBD organisms was not available for the remaining for 18 dogs.

#### 4.1.2. Human Serum Samples

To evaluate the diagnostic utility of *B. henselae* rPap31 and its recombinant fragments (rPap31-NTD, rPap31-MD, and rPap31-CTD) for the serodiagnosis of human Bartonelloses, a set of 36 diagnostic serum samples obtained from the repository maintained by the NCSU-CVM-IPRL (NCSU IRB approval# 1960) were tested by ELISA. Detailed methods for the *Bartonella* PCR, BAPGM enrichment blood culture, and the IFA serological panel used to test these study participants were previously published [19,42]. Group III consisted of 18 sera from humans naturally exposed to *Bartonella* spp. All 18 individuals were *B. henselae, B. koehlerae*, or *B. vinsonii* subsp*. berkhoffii* genotype III seropositive. Fifteen Group III humans were *B. henselae* seropositive (IFA titer of ≥1:64), Table 2 and Supplementary Table S1. The remaining three individuals were *B. koehlerae* and *B. vinsonii* subsp*. berkhoffii* genotype III IFA seropositive, but were *B. henselae* seronegative. Of the 18 people, 8 were IFA seropositive to *B. vinsonii* subsp*. berkhoffii* genotype I, 10 to *B. vinsonii* subsp*. berkhoffii* genotype II, 14 to *B. vinsonii* subsp*. berkhoffii* genotype III, and 11 to *B. koehlerae.* A total of 5 of the 18 people were seropositive to all 5 IFA antigens *B. henselae, B. koehlerae*, and *B. vinsonii* subsp*. Berkhoffii* genotypes I, II, and III.

Based on *Bartonella* blood qPCR, or droplet digital PCR (ddPCR) [43] and IFA serology, 14 of 18 Group III humans were *Bartonella* PCR-positive and *B. henselae* IFA-positive (IFA titer of ≥1:64. A total of 2 of 18 were *Bartonella* PCR-positive, but *B. henselae* IFA seronegative, and two were *B. henselae* IFA-positive and *Bartonella* PCR-negative. Of the 16 *Bartonella* PCR-positive humans, 6 were infected with *B. henselae* (blood DNA extraction) and *B. henselae* IFA-positive (IFA titer of ≥1:64); 6 were *Bartonella* ddPCR-positive (DNA sequencing for species determination was not technically possible) and *B. henselae* IFA seropositive; and 1 *B. henselae* seropositive individual was infected with *B. vinsonii* subsp*. berkhoffii* genotype I. The three remaining people were *Bartonella* ddPCR-positive, *B. henselae* IFA-negative, but were seropositive to both *B. koehlerae* and *B. vinsonii* subsp*. berkhoffii* genotype III.

To evaluate ELISA rPap31 proteins for specificity, Group IV sera (n = 18) were selected from the study participants that were *Bartonella* spp. IFA-negative and were *Bartonella* PCR-negative from blood and BAPGM enrichment blood culture, Table 2 and Supplementary Table S1. A total of 9 of these 18 human sera were from healthy donors tested in a previous study [19]. All Group IV human sera were nonsereoreactive (IFA titer of ≤1:16) to *B. henselae*, *B. vinsonii* subsp*. berkhoffii* genotypes I, II, and III, and *B. koehlerae*. Based on the *Bartonella* PCR and BAPGM enrichment culture testing, *Bartonella* DNA was not amplified from any blood or BAPGM enrichment blood culture.

### 4.2. In Silico Analysis of Bartonella henselae Pap31

The amino acid sequence of the *B. henselae* Pap31 protein (1 to 279 amino acids) was derived from the NCBI database. To determine the signal peptide and its cleavage site, signal peptides of the Pap31 protein were determined using the Signal P-5.0 Server (http://www.cbs.dtu.dk/services/SignalP/ accessed on 8 June 2020). The transmembrane topology of *B. henselae* was examined by TMHMM.v2.0 (http://www.cbs.dtu.dk/services/TMHMM/ accessed on 8 June 2020). PSORTb v3.0.3 was used to predict the Pap31 subcellular localization site of Pap31 (https://www.psort.org/psortb/ accessed on 8 June 2020). The integration and visualization of annotated and predicted protein sequence features of *B. henselae* Pap31 were predicted using Protter (https://wlab.ethz.ch/protter/start/ accessed on 8 June 2020).

### 4.3. Amplification of B. henselae pap31 Gene and pap31 Gene Fragments: N-Terminal Domain (NTD), Middle Domin (MD), and C-Terminal Domain (CTD)

The entire *B. henselae* San Antonio (*Bh* SA2) Pap31 (rPap31) protein and each of the three Pap31 fragments (C-terminal-, middle, and N-terminal domains of Pap31) were cloned and expressed using the *Escherichia coli* expression system. Four Pap31 primer sets were used for conventional PCR amplification, as shown in Table 3. Conventional PCR was performed in a 25 μL final reaction volume containing 12.5 μL of Q5 High-Fidelity 2X Master mix (New England Biolabs, Ipswich, MA, USA, cat. No. M0492S); 0.2 μL of 100 μM of each forward and reverse primer (IDT-DNA Technology); 7.3 μL of molecular-grade water; and 5 μL of DNA from each sample tested. A total of 5 μL of Ultra-Pure, molecular grade water (Genesee Scientific, San Diego, CA, USA) and 5 μL of DNA extracted from *Escherichia coli* were used as negative controls. Genomic DNA from *B. henselae* Houston-1 was used as a positive control. For all 4 PCR assays, conventional PCR was performed in an Eppendorf Mastercycler EP gradient under the following conditions: a single hot-start cycle at 95 °C for 2 min followed by 30 cycles of denaturing at 95 °C for 30 s, annealing at 55 °C for 30 s, and extension at 72 °C for 30 s. Amplification was completed by an additional cycle at 72 °C for 2 min. The PCR products were analyzed by 2% agarose gel electrophoresis with detection using gel red (Thermo Scientific, Rockford, IL, USA). Prior to the ligation reaction for cloning, PCR products were purified by gel extraction using PureLink quick gel extraction and a PCR purification combo kit (Invitrogen, Carlsbad, CA, USA).

### 4.4. Cloning of B. henselae Recombinant pap31 Protein and pap31 Gene Fragments: N-Terminal Domain (NTD), Middle Domain (MD), and C-Terminal Domain (CTD)

The *pap31* gene was cloned and expressed using a Champion^TM^ pET200 Directional TOPO^®^ Expression kit (Invitrogen, Carlsbad, CA, SUA). Whole *pap31* (encoding 25 to 279 amino acids (aa)) and 3 fragments of the *pap31* gene, C-terminal (25 to 94 aa), middle (95 to 187 aa), and N-terminal (188 to 279 aa) domains, were cloned according to the manufacturer’s instructions (Invitrogen, Carlsbad, CA, USA), Table 3.

Recombinant plasmids were subsequently transformed into *E. coli* BL21 (DE3) Star™ chemically competent cells and plated on LB plates containing 50 µg/mL of kanamycin. Positive clones were confirmed by plasmid extraction followed by sequencing. Amplicon products were sequenced using T7 and T7 reverse primers by Sanger sequencing (Genewiz, Research Triangle Park, NC, USA). Chromatogram evaluation and sequence alignment was performed using SnapGene software (GSL Biotech; available at snapgene.com; accessed on 8 June 2020) to confirm the in-frame cloning of *pap31* and *pap31* gene fragments. The nucleotide sequence homology of the PCR amplicon was performed with available nucleotide sequences at the NCBI databases using the NCBI BLAST program (v2.0).

### 4.5. Expression and Purification of Recombinant Pap31 and Pap31 Domains (N-Terminal, Middle, and C-Terminal Domains)

To confirm and monitor the expression of recombinant proteins, each *E. coli* BL21 (DE3) clone containing *pap31* inserts was grown in 25 mL of LB broth at 37 °C containing 50 µg/mL of kanamycin. When the optical density (OD_600 nm_) of the culture bacteria reached 0.6 to 0.8, 1mM IPTG was added to induce protein expression. Cultures were examined for protein expression at 0, 3, 6, and 8 h post-IPTG induction at 30 °C by immunoblotting of whole cell lysates using a Pierce™ 6X-His Epitope-Tag mouse monoclonal antibody (Thermo Scientific, Rockford, IL, USA) and an anti-mouse IgG secondary antibody (Rockland, Gilbertsville, PA, USA).

Recombinant rPap31s (rPap31, rPap31-NTD, rPap31-MD, and rPap31-CTD) were purified by column-chromatography using HisPur^TM^ Cobalt spin columns, according to the manufacturer’s instructions (Thermo Scientific, Rockford, IL, USA). For purification, each recombinant clone was grown at 37 °C overnight in a 25 mL LB broth containing 50 µg/mL of kanamycin in a shaker at 200 rpm. After overnight incubation, 5ml of the overnight culture was transferred to fresh 1000 mL LB broth containing 50 µg/mL of kanamycin and was incubated at 37 °C for 2 h in a shaking platform at 200 rpm. After 2 h, IPTG was added to a final concentration of 1 mM and incubated at 30 °C for 8 h. The cultures were then centrifuged at 5000× *g* to obtain the pellets. The pellets were processed using BugBuster Master Mix (EMD Millipore Corp., Billerica, MA, USA) to purify inclusion bodies according to the manufacturer’s instructions. After the inclusion body purification, the pellets were then subsequently solubilized in 1× PBS containing 6 M of urea. His-tagged proteins were extracted from inclusion body suspensions using HisPur™ Cobalt spin columns (Thermo Scientific, Rockford, IL, USA). Wash buffer (1× PBS with 6 M urea and 5 mM imidazole) and Elution buffer (1× PBS with 6 M urea and 100 mM imidazole) were used during the His spin column purification steps. Eluted proteins were analyzed by SDS-PAGE in Criterion™ using 4–15% gradient polyacrylamide tris-glycine precast midi gels (Bio-Rad, Hercules, CA, USA) at a constant current (100 V) for 1 h 50 min in a 1× running buffer (25 mM tris, 192 mM glycine, 0.1% SDS). A pre stained broad-range (10–250 kDa) molecular weight protein marker (Bio-Rad, Hercules, CA, USA) was used as a standard. Fractionated proteins were visualized by staining the gel overnight with Bio-safe™ Coomassie brilliant blue (Bio-Rad, Hercules, CA, USA). Purified recombinant proteins were verified by WB using Pierce™ 6X-His Epitope-Tag mouse monoclonal antibody (Thermo Scientific, Rockford, IL, USA) and anti-mouse IgG secondary antibody (Rockland, Gilbertsville, PA, USA). Proteins were dialyzed into phosphate-buffered saline (PBS; overnight) using Slide-A-Lyzer™ Dialysis Cassettes, (2–10 kDa MWCO cutoff; Thermo Scientific, Rockford, IL, USA).

### 4.6. Evaluate the Sensitivity and Specificity of B. henselae Recombinant Proteins Recombinant Pap31 and Pap31 Domains (N-Terminal, Middle, and C-Terminal Domains) by ELISA

The sensitivity and specificity of purified recombinant Pap31 proteins were evaluated by ELISA using sera from Group I (n = 36) and II (control group; n = 34) dogs, and from Group III (n = 18) and IV (control group; n = 18) humans, as described in the Methods Sections for canine and human Bartonelloses, respectively. Each protein was immobilized in duplicate in ELISA plate wells (500 ng/well), using carbonate buffer and standard methods that have been well described [44]. Sera from naturally infected and unexposed (control) dogs and humans with *Bartonella* spp. were used to screen the purified recombinant proteins. In brief, ELISA plate wells were coated with 100 µL of 10 µg/mL recombinant purified proteins using a carbonate buffer, pH 9.6. Plates were incubated at 4 °C overnight. After overnight coating, plates were washed with 1× PBS 4 times. Plates were then incubated with a blocking solution (1× PBS containing 3% milk) at room temperature (RT) for 2 h, followed by washing with 1× PBS containing 0.05% Tween-20 (Bio-Rad, Hercules, CA, USA) 4 times. Plates were incubated with 100 µL of dog or human sera at 1:100 dilution in 1× PBS containing 3% milk at RT for 1 h. Plates were washed with 1× PBS containing 0.05% Tween-20 4 times, followed by incubation with 100 µL of secondary antibody for 1 h at RT. HRP-conjugated goat anti-dog IgG (1:2,000 dilution; Invitrogen, Carlsbad, CA, USA) and HRP-conjugated goat anti-human IgG (1:5,000; Abcam, Cambridge, MA, USA) in 1× PBS containing 3% milk were used as secondary antibodies for dog and human ELISA testing, respectively. After secondary antibody incubation, the plates were washed 5 times with 1× PBS containing 0.05% Tween. A total of 50 µL of 1-Step™ Ultra TMB-ELISA substrate solution (Invitrogen, Carlsbad, CA, USA) was then added to the plate wells and incubated at RT for 15 min, followed by the addition of 2M H_2_SO_4_ to stop the reaction. The absorbance was measured at 450 nm of the wavelength using a Tecan plate reader. Sera from dogs naturally infected with *B. henselae* (*B. henselae* IFA titer = 1: 512) and *Bartonella* PCR-negative and IFA-negative dogs were used as the positive and negative controls, respectively. The plate wells coated with only coating buffer (without protein) were used as blanks to determine the subtract background noise. Based on the results from the negative controls, a baseline was established for scoring individual samples as positive or negative for infection with *Bartonella*. The average absorbance value was calculated for each set of duplicate samples.

### 4.7. Development of Pap31 B-Cell Epitope-Based ELISA for Bartonelloses

**Figure 9:**
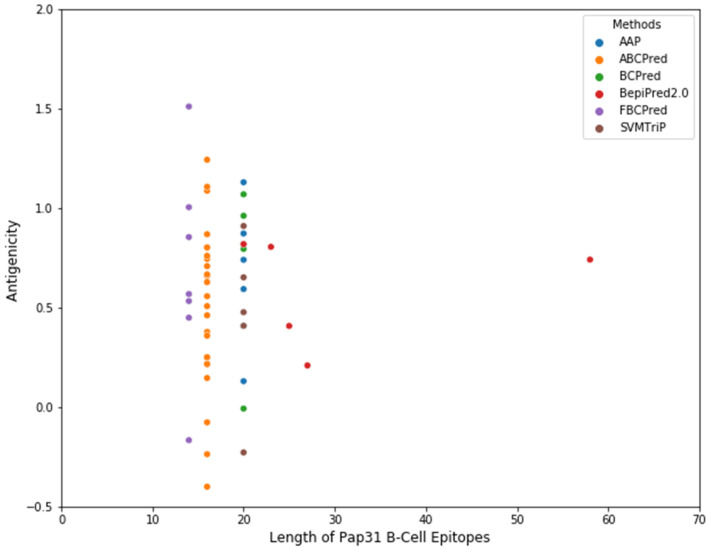
A scatter plot showing the number of predicted B-cell epitopes of *B. henselae* Pap31. Six algorithms (AAP, ABCPred, BCPred, BepiPred 2.0, FBCPred, and SVMTriP) were employed to predict the linear B-cell epitopes of *B. henselae* Pap31. A total of 53 linear B-cell epitopes of variable lengths were predicted by these methods: AAP (n = 6); ABCPred (n = 25); BCPred (n = 5), BepiPred 2.0 (n = 5) FBCPred (n = 7), and SVMTrip (n = 5).

Several B-cell epitope prediction tool methods have been developed over the years, based on a Hidden Markov Model (HMM) with an amino acid propensity scale, Neural Networks, Support Vector Machines, and SVM models trained on the frequency of amino acid pairs (AAPs) [45,46,47]. BepiPred 2.0 with default parameter settings provided by IEDB (Immune Epitope Database and Analysis Resource) were applied to the *B. henselae* Pap31 protein sequence to predict the linear B-cell epitopes [45,48]. Subsequently, five additional algorithms, AAP [49], ABCPred [50], BCPreds [49], FBCPred [51], and SVMTriP [52], were also employed to predict the linear B-cell epitopes of *B. henselae* Pap31, Supplementary Table S2. The molecular weight, amino acid composition, estimated lifetime, and grand average of hydrophobicity (gravy) was determined using the Expasy ProtParam program (https://web.expasy.org/protparam/ accessed on 20 January 2020). The protein secondary structure of *B. henselae* Pap31 was determined using PSIPRED (http://bioinf.cs.ucl.ac.uk/psipred/ accessed on 22 January 2020). The antigenicity of the predicted linear B-cell epitopes was evaluated by VaxiJen 2.0. A total of 53 linear B-cell epitopes were predicted by the above-mentioned 6 algorithms, Figure 9 and Supplementary Table S2. Based on the predicted linear B-cell epitopes, membrane topology, and antigenicity scores (>0.75), four highly antigenic Pap31 B-cell epitopes (P1, P2, P3, and P4) were selected and synthesized using a commercial company (GenScript, Piscataway, NJ, USA), Table 4.

To assess the antigenicity and seroreactivity of four Pap31 B-cell epitopes for canine Bartonelloses, Pap31 B-cells epitopes were tested by ELISA and dot blot. Dog ELISA was performed using three serum samples from three experimentally inoculated dogs, five *B. henselae* IFA-positive Group I dogs (IFA titer of ≥1:256), and four Group II dogs (*Bartonella* PCR-negative and IFA-negative). Twenty-eight-day post-inoculation sera from two dogs that were infected subcutaneously with *B. henselae* SA2 and one dog infected with *B. vinsonii* subsp*. berkhoffii* genotype III [53], were obtained from NCSU-CVM-IPRL. A total of 1 sera collected on post-inoculation day 21 from a dog intravenously inoculated with *B. henselae* CSU 1 strain was kindly provided Dr. Michael Lappin, the Department of Clinical Sciences, Colorado State University. For human testing, sera from eight Group III *B. henselae* IFA-positive (IFA titer of ≥1:128) and four Group IV (control) humans were used. Of the eight *B. henselae* IFA-positive human samples, two serum samples were from *B. henselae* PCR-positive humans.

ELISA was performed, as described above in the Methods Section, with minor modifications. ELISA plate wells were coated with 100 µL of 10 and 20 µg/mL peptides using a carbonate buffer. Sera from dogs and humans were used at two dilutions (1:50 and 1:100 dilution). Secondary antibodies were used as described above in the Methods Section. Dot blot was performed as follows: peptides at a concentration of 15 μg and 20 μg were spotted onto PVDF membranes and air dried. The strips were blocked overnight with 5% nonfat dry milk in 1× TBS containing 0.05% Tween-20 (TBST). After overnight blocking, each strip containing the peptides was incubated with 2 dilutions (1:50 and 1:100) of primary antibody (serum samples from naturally exposed dogs (*Bartonella* IFA-positive dogs; n = 8) and humans (*Bartonella* IFA-positive n = 8) with a *B. henselae* IgG titer of ≥1:128) in 1× TBST containing 3% nonfat dry milk for 1 h. A negative-control dog or negative human serum sample (*B. henselae*, *B. vinsonii* subsp*. berkhoffii*, and *B. koehlerae* IgG IFA titers < 1:16) were chosen from the VBDDL or IPRL archives, respectively. After washing with 1× TBST, a secondary antibody (alkaline phosphatase (AP)-conjugated goat anti-dog whole IgG or AP-conjugated anti-human) was added at serial dilutions (1:5000 to 1:10,000) in 1× TBS-T containing 3% nonfat dry milk, and then incubated at room temperature for an hour. After washing, the membrane strips were developed using a commercially available substrate solution containing nitroblue tetrazolium chloride (NBT) and 5-bromo-4-chloro-3′-indolyphosphate p-toluidine salt (BCIP; Pierce, Rockford, IL, USA). Image acquisition was performed using a ChemiDoc imaging system (Bio-Rad, Hercules, CA, USA). The blots were analyzed with Image Lab software (v4.1; Bio-Rad, Hercules, CA, USA).

### 4.8. Statistical Analysis

To determine the sensitivity, specificity, and cutoff values for ELISA, the receiver operating characteristic (ROC) analysis was performed with 95% Cis, as previously described [54,55]. Since IFA is considered as a gold-standard assay for the diagnosis of canine and human Bartonelloses, the IFA results were considered as evidence of the exposure to *Bartonella* spp. for the calculation of the sensitivity and specificity of ELISA in this study. The optimal density (OD) cutoff values were determined to maximize the Youden index, as previously described (53). The Youden index is the metric for assessing the performance of a diagnostic test. The index is defined by Equation (1), and a, b, c, and d in (1), respectively, denote the numbers of true positives, false negatives, false positives, and true negatives. J = 0 represents a diagnostic test, which gives the same proportion of positives for both the control and infected groups, and J = 1 represents a diagnostic test with no false positive and false negative detected. The optimal cutoff value is the one that maximizes the *J*.

(1) $$\mathrm{choose}\;\mathrm{cutoff}\;\mathrm{subjected}\;\mathrm{to}\;\max\;J=\frac{\left(ad-bc\right)}{\left(a+b\right)\left(c+d\right)}$$

To compare the agreement between the ELISA and *Bartonella* IFA testing results, the positive, negative, and overall percent agreement between ELISA and IFA assays were calculated as previously described [56]. To measure the level of agreement between the *Bartonella* IFA and ELISA tests, the kappa statistic was calculated as previously described [57]. Differences in the IgG reactivity to target proteins between the infected and control groups were analyzed using the Mann–Whitney U test. The scatterplots and ROC curve analysis for ELISA seroreactivity were generated in the Windows 10 operating system with the help of the Anaconda Navigator, v1.9.12. The scatter plots were generated using Python 3. 6.13 in Jupyterlab 3.2.1 and the ROC curves were analyzed using R v3.6.1 in R studio 1.1.456 (accessed on November 25, 2021). The *p*-values of less than 0.05 were considered statistically significant.

## Figures and Tables

**Table 1:** Comparison of *Bartonella* IFA- and recombinant Pap31-based ELISA results for dog and human sera.

Dog Groups (I and II)				Human Groups (III and IV)			
ELISA	IFA		PPA, NPA, and OPA (Agreement ELISA/IFA)	ELISA	IFA		PPA, NPA, and OPA (Agreement ELISA/IFA)
	POS (n = 36)	NEG (n = 34)			POS (n = 18)	NEG (n = 18)	
rPap31 ELISA				rPap31 ELISA			
Positive	15	2	42%, 94%, 67%	Positive	13	7	72%, 61%, 67%
Negative	21	32		Negative	5	11	
rPap31-NTD ELISA				rPap31-NTD ELISA			
Positive	33	15	92%, 56%,74%	Positive	16	8	89%, 56%, 72%
Negative	3	19		Negative	2	10	
rPap31-MD ELISA				rPap31-MD ELISA			
Positive	25	8	69%, 76%, 73%	Positive	18	15	100%, 17%, 58%
Negative	11	26		Negative	0	3	
rPap31-CTD ELISA				rPap31-CTD ELISA			
Positive	29	16	81%, 53%, 67%	Positive	1	0	6%, 100%, 53%
Negative	7	18		Negative	17	18	

Group I dogs (n = 36) were naturally infected with *Bartonella* spp. All Group I dogs were *B. henselae* IFA-positive (IFA titer ≥ 1:64). Group II dogs consisted of 34 *Bartonella* spp. IFA-negative and PCR-negative control dogs. Group III humans (n = 18) were naturally infected with *Bartonella* spp. Group IV consisted of 18 *Bartonella* PCR, culture, and IFA-negative humans. PPA = positive percentage (%) agreement; NPA = negative % agreement; and OPA = overall % agreement between *Bartonella* spp. IFA and ELISAs.

**Table 2:** *Bartonella* testing results for dog and human clinical samples used for comparative ELISA testing in this study.

Samples Used for ELISA Testing						
Groups ID	Group Info	Bart. IFA (n)	Bart. PCR (Combined qPCR or ddPCR or BAPGM)	Bart. qPCR n (Strain)	Bart. ddPCR	BAPGM Enrichment Culture (n)
**Dogs Serum Samples**						
Group I (n = 36)	*Bartonella* spp. naturally infected dogs (Bart. IFA POS)	POS (36 *)	(POS) 3; NEG (33)	2 (Bv); 1 (Bh); NEG (33)	n/a	NEG (8); n/a (28)
Group II (n = 34)	*Bartonella* spp. PCR NEG and IFA NEG dogs (control)	NEG (34)	NEG (34)	NEG (34)	n/a	NEG (21); n/a (13)
**Human Serum Samples**						
Group III (n = 18)	*Bartonella* spp. naturally infected humans (Bart. IFA-POS)	POS (18; 15 *)	POS (16); NEG (2)	5 (Bh); 1 (BvbTI); NEG (12)	POS (12); NEG (1); n/a (5)	POS (9); NEG (9)
Group IV (n = 18)	*Bartonella* spp. PCR-NEG and IFA-NEG humans (control)	NEG (18)	n/a	NEG (18)	n/a	NEG

* represents dogs or humans that were IFA-positive for *B. henselae.* IgG titers of ≥1:64 were considered positive for *Bartonella* exposure. POS = positive; NEG = negative; n/a = not available; *Bh = B. henselae*; Bv = *B. vinsonii*; BvbTI = *B. vinsonii* subsp. *berkhoffii* genotype I; Bart. = *Bartonella* spp.; and ddPCR = droplet digital PCR.

**Table 3:** List of PCR primers used for the cloning, expression, and purification of the *Bartonella henselae* San Antonio 2 pap31 gene using a Champion™ pET200 Directional TOPO^®^ Expression kit.

Recombinant Proteins	Selected Region of Pap31 for Cloning and Purification (NCBI Reference Sequence; Selected Amino Acids (aa))	Mol. Wt. (kDa)	Primers Used for PCR Amplification and Cloning (Sequence 5′ → 3′)
Whole Pap31 (rPap31)	CDO39660.1; 25 to 279 (255 aa)	27.52	Pap31-73F CACCGTTATCGTTCCTCATGAAGTAGCG
			Pap31-837R GAATTTGTACGCTACACCAACAC
N-terminal domain (rPap31NTD)	CDO39660.1; 25 to 94 (70 aa)	7.46	Pap31-73F CACCGTTATCGTTCCTCATGAAGTAGCG
			Pap31-282R AAGATCCATGTTGGAACCTGCATA
Middle domain (rPap31-MD)	CDO39660.1; 95 to 187 (93 aa)	10.01	Pap31-283F CACCGGAAATAATATGATTCTAGGAGTTGA
			Pap31-561R AGCAACATAAGGCATAATGCGATC
C-terminal domain (rPap31-CTD)	CDO39660.1; 188 to 279 (92 aa)	10.08	Pap31-562F CACCGGTGGTGTTTCCTATGCACAGGTA
			Pap31-837R GAATTTGTACGCTACACCAACAC

Whole Pap31 (rPap31), N-terminal domain (NTD), middle domain (MD), and C-terminal domain (CTD) of rPap31 of *B. henselae* were cloned, expressed, and purified using the *E. coli* expression system. The four nucleotides in bold represents nucleotides that were added at the 5′ end of the forward primer to enable directional cloning in the pET200/D-TOPO vector. Mol. wt. = molecular weight; aa = amino acids.

**Table 4:** Selected highly antigenic B-cell epitopes of *B. henselae* Pap31 for ELISA testing. Six algorithms were used to predict the linear B-cell epitopes. Of the 53 predicted B-cell epitopes identified by the 6 algorithms, 4 highly antigenic B-cell epitopes (antigenicity score > 0.75) were selected based on antigenicity, membrane topology, sequence homology, and protein secondary structure.

Pap31 B-Cell Epitopes ID	Selected Linear B-Cell Epitopes of *B. henselae* Pap31	Location (NCBI Reference Number: CDO39660.1; aa)	Antigenicity	Hydrophobicity	Hydropathicity	Hydrophilicity	pI	Mol wt (Da)
P1	VETDAVWADREDAKTSSAEA	102 to 121	1.0155	−0.25	−0.79	0.74	4	2151.5
P2	AQGKTSDNVAAVDKHT	142 to 157	0.8856	−0.25	−0.86	0.43	7.1	1642
P3	GFTLGGGVDFAMTDNV	226 to 241	1.1084	0.1	0.5	−0.39	3.6	1601
P4	KKFEKEGSEFSYKTND	255 to 270	1.0881	−0.43	−1.97	1.07	6.6	1937.3

## Data Availability

Data supporting the conclusions of the authors are included in the article. To assure participant confidentiality, please contact E.B.B. for questions relative to the raw data.

## Supplementary Materials

The following supporting information can be downloaded at: https://www.mdpi.com/article/10.3390/pathogens11020182/s1, Table S1: *Bartonella* and canine vector-borne disease (CVBD) testing results for dogs and humans clinical samples used for comparative ELISA testing in this study and Table S2: The full list of linear B-cell epitopes of *Bartonella henselae* Pap31.
