# Supplementary material for: Bartonella henselae Recombinant Pap31 for the Diagnosis of Canine and Human Bartonelloses
Source: Pathogens. 2022 Jan 28;11(2):182. doi: 10.3390/pathogens11020182 (PMC8877253; doi:10.3390/pathogens11020182)
Supplement: Supplementary file 1 [file pathogens-11-00182-s001.zip › pathogens-1556388-supplementary.pdf]

Supplementary materials

Supplementary Table S1. *Bartonella* and canine vector-borne disease (CVBD) testing results for dogs and humans clinical samples used for comparative ELISA testing in this study. POS= positive; NEG= negative; NT= not tested; n/a = not available; *Bh* = *B. henselae* ; *Bvb* TI= *B. vinsonii* subsp. *berkhoffii* (*Bvb*) genotype I; *Bvb* TII= *Bvb* genotype II; *Bvb* TIII= *Bvb* genotype III; *Rr* = *Rickettsia rickettsii; Bart* .= *Bartonella* spp., ddPCR= droplet digital PCR; IFA= Immunofluorescent antibody assay; CVBD PCR = PCR testing for *Babesia* , *Ehrlichia* , *Anaplasma* , *Rickettsia* , hemotropic *Mycoplasma* and *Leishmania* spp.; CVBD serology = IFA testing for *Rickettsia rickettsii, Ehrlichia canis, Babesia canis,* and *Babesia gibsoni* plus ELISA testing (SNAP 4Dx PLUS ELISA, IDEXX Laboratories, Westbrook, Maine) for *Anaplasma phagocytophilum, Anaplasma platys, Borrelia burgdorferi, Ehrlichia canis, and Ehrlichia ewingii.* Detailed methods for the *Bartonella* PCR, BAPGM (*Bartonella* Alpha Proteobacteria Growth Medium) enrichment blood culture, and the IFA serological panel used to test these study participants have been published previously [14,21]. *Bartonella* blood droplet digital PCR (ddPCR) was perfomred as described previously [43]. Of the four dogs positive for CVBD by serology, one dog was seropositive to *Babesia canis* (IFA titer 1:1024) and *Babesia gibsoni* (IFA titer 1:4096), one dog was seropositive to *Ehrlichia canis* (IFA titer 1:2048), one dog was seropositive to *Babesia canis* (IFA titer 1:2048) and *Babesia gibsoni* (IFA titer 1:2048), and the remaining dog was seropositive to *Babesia canis* (IFA titer 1:64). Two (2*) of these four dogs were also *R. rickettsii* IFA positive.

**Testing**	**Testing Results**	**Testing Reults for Dogs and Humans Groups**			
		**Group I (*Bartonella* infected dogs; n=36)**	**Group II (control dogs; n=34)**	**Group III (*Bartonella* infected humans; n=18)**	**Group IV (control humans; n=18)**
Bh IFA	POS	36	0	15	0
	NEG	0	34	3	18
Bvb TI IFA	POS	23	0	8	0
	NEG	13	34	10	18
Bvb TII IFA	POS	n/a	n/a	10	0
	NEG	n/a	n/a	8	18
Bvb TIII IFA	POS	n/a	n/a	14	0
	NEG	n/a	n/a	4	18
Bk IFA	POS	32	0	11	0
	NEG	4	34	7	18
Bart. PCR	POS	3	0	6	0
	NEG	33	34	12	18
Bart. ddPCR	POS	n/a	n/a	12	n/a
	NEG	n/a	n/a	1	n/a
	NT	n/a	n/a	5	n/a
BAPGM	POS	0	0	9	0
	NEG	8	21	9	18
	NT	28	13	0	0
Rr IFA	POS	2*	10	n/a	n/a
	NEG	24	6	n/a	n/a
	NT	10	18	n/a	n/a
CVBD PCR	POS	0	0	n/a	n/a
	NEG	24	16	n/a	n/a
	NT	12	18	n/a	n/a
CVBD Serology	POS	4	0	n/a	n/a
	NEG	24	16	n/a	n/a
	NT	8	18	n/a	n/a

Supplementary Table S2. The full list of linear B-cell epitopes of *Bartonella henselae* Pap31. Six algorithms (AAP, ABCPred, BCPred, BepiPred 2.0, FBCPred, and SVMTriP) were employed to predict linear B-cell epitopes of *B. henselae* Pap31. The antigenicity of predicted linear B-cell epitopes was determined by VaxiJen 2.0.

Start	End	Peptide	Peptide Length	Methods	Antigenicity	Hydrophobicity	Hydropathicity	Hydrophilicity	Charge	pI	Mol Wt. (Da)	Topology
219	238	DKTKTLVGFTLGGGVDFAMT	20	AAP	0.5939	0.01	0.31	-0.15	0	6.31	2058.68	outside
141	160	FAQGKTSDNVAAVDKHTDSL	20	AAP	0.7412	-0.19	-0.57	0.3	-0.5	5.31	2104.54	outside
62	81	TDPNKKDKLFSKDDTPKPSG	20	AAP	0.1313	-0.44	-1.93	1.14	1	8.71	2218.72	outside
250	269	SDFGKKKFEKEGSEFSYKTN	20	AAP	0.8734	-0.38	-1.69	0.9	1	8.66	2356.85	outside
102	121	VETDAVWADREDAKTSSAEA	20	AAP	1.1311	-0.25	-0.79	0.74	-4	4.02	2151.48	outside
32	51	APTVISAPAFSWTGFYIGGQ	20	AAP	0.4094	0.16	0.55	-0.86	0	5.88	2070.62	outside
83	98	MGGIYAGSNMDLGNNM	16	ABCPred	0.2473	0	-0.12	-0.4	-1	3.8	1645.1	outside
58	73	KVEITDPNKKDKLFSK	16	ABCPred	0.2149	-0.39	-1.33	1.03	2	9.43	1890.44	outside
209	224	DAEIATAQLFDKTKTL	16	ABCPred	-0.2359	-0.15	-0.22	0.29	-1	4.56	1765.22	outside
255	270	KKFEKEGSEFSYKTND	16	ABCPred	1.0881	-0.43	-1.97	1.07	0	6.59	1937.31	outside
243	258	LRAEYRYSDFGKKKFE	16	ABCPred	0.6593	-0.43	-1.49	0.78	2	9.43	2037.52	outside
100	115	LGVETDAVWADREDAK	16	ABCPred	0.802	-0.22	-0.64	0.68	-3	4.11	1775.12	outside
140	155	AFAQGKTSDNVAAVDK	16	ABCPred	0.558	-0.16	-0.33	0.3	0	6.31	1621.98	outside
119	134	AEAIGQDELETFRDSL	16	ABCPred	0.147	-0.2	-0.56	0.57	-4	3.84	1794.13	outside
179	194	ADRIMPYVAGGVSYAQ	16	ABCPred	0.2178	-0.02	0.18	-0.36	0	6.18	1698.15	outside
32	47	APTVISAPAFSWTGFY	16	ABCPred	0.379	0.17	0.68	-0.98	0	5.88	1715.15	outside
187	202	AGGVSYAQVQAVSSTK	16	ABCPred	0.8694	-0.05	0.12	-0.28	1	8.94	1552.93	outside
163	178	KEKWSGATRVRIGFTA	16	ABCPred	0.7092	-0.24	-0.57	0.27	3	11.01	1807.31	outside
262	277	SEFSYKTNDFRVGVAY	16	ABCPred	1.2438	-0.17	-0.52	-0.04	0	6.41	1883.26	outside
226	241	GFTLGGGVDFAMTDNV	16	ABCPred	1.1084	0.1	0.5	-0.39	-2	3.57	1601	outside
40	55	AFSWTGFYIGGQVGNF	16	ABCPred	-0.0756	0.16	0.41	-1.04	0	5.88	1751.17	outside
202	217	KVTQAADDAEIATAQL	16	ABCPred	0.746	-0.12	-0.08	0.25	-2	4.03	1645.02	outside
17	32	ASAAQAADVIVPHEVA	16	ABCPred	0.6289	0.07	0.74	-0.21	-1.5	4.36	1548.93	outside
89	104	GSNMDLGNNMILGVET	16	ABCPred	0.7611	-0.02	-0.01	-0.19	-2	3.67	1665.12	outside
70	85	LFSKDDTPKPSGFMGG	16	ABCPred	-0.3994	-0.12	-0.64	0.26	0	6.31	1684.12	outside
9	24	TSVIALISASAAQAAD	16	ABCPred	0.3597	0.1	1.11	-0.39	-1	3.8	1488.87	outside
132	147	DSLKKANAAFAQGKTS	16	ABCPred	0.4619	-0.23	-0.69	0.39	2	9.72	1637.04	outside
150	165	VAAVDKHTDSLALKEK	16	ABCPred	0.8036	-0.21	-0.34	0.58	0.5	7.1	1725.2	outside
126	141	ELETFRDSLKKANAAF	16	ABCPred	0.2521	-0.25	-0.57	0.5	0	6.53	1840.28	outside
171	186	RVRIGFTAADRIMPYV	16	ABCPred	0.5082	-0.13	0.29	-0.13	2	10.75	1865.45	outside
48	63	IGGQVGNFSSKVEITD	16	ABCPred	0.6677	-0.05	-0.08	0.03	-1	4.38	1651.05	outside
244	263	RAEYRYSDFGKKKFEKEGSE	20	BCPred	0.9622	-0.49	-1.98	1.18	1	8.66	2454.95	outside
74	93	DDTPKPSGFMGGIYAGSNMD	20	BCPred	-0.0066	-0.1	-0.67	0.14	-2	3.94	2060.52	outside
24	43	DVIVPHEVAPTVISAPAFSW	20	BCPred	0.8199	0.14	0.79	-0.56	-1.5	4.36	2135.73	outside
49	68	GGQVGNFSSKVEITDPNKKD	20	BCPred	0.7957	-0.26	-1.1	0.57	0	6.46	2120.6	outside
107	126	VWADREDAKTSSAEAIGQDE	20	BCPred	1.0702	-0.27	-1.03	0.78	-4	4.02	2178.52	outside
24	43	DVIVPHEVAPTVISAPAFSW	20	BepiPred2.0	0.8199	0.14	0.79	-0.56	-1.5	4.36	2135.73	outside
55	81	FSSKVEITDPNKKDKLFSKDDTPKPSG	27	BepiPred2.0	0.2101	-0.34	-1.34	0.87	1	8.66	3009.74	outside
107	164	VWADREDAKTSSAEAIGQDELETFRDSLKKANAAFAQGKTSDNVAAVDKHTDSLALKE	58	BepiPred2.0	0.742	too long	too long	too long	too long	too long	too long	outside
198	222	VSSTKVTQAADDAEIATAQLFDKTK	25	BepiPred2.0	0.4086	-0.18	-0.34	0.35	-1	4.69	2639.26	outside
247	269	YRYSDFGKKKFEKEGSEFSYKTN	23	BepiPred2.0	0.806	-0.4	-1.78	0.71	2	9.2	2839.43	outside
76	89	TPKPSGFMGGIYAG	14	FBCPred	-0.1657	0.06	-0.04	-0.39	1	8.94	1382.8	outside
108	121	WADREDAKTSSAEA	14	FBCPred	1.5116	-0.35	-1.31	0.91	-2	4.32	1536.74	outside
253	266	GKKKFEKEGSEFSY	14	FBCPred	0.8555	-0.37	-1.73	1.02	1	8.71	1664.04	outside
57	70	SKVEITDPNKKDKL	14	FBCPred	0.45	-0.41	-1.44	1.14	1	8.76	1615.06	outside
24	37	DVIVPHEVAPTVIS	14	FBCPred	0.534	0.11	0.91	-0.34	-1.5	4.36	1475.91	outside
146	159	TSDNVAAVDKHTDS	14	FBCPred	1.0056	-0.26	-0.86	0.54	-1.5	4.42	1459.67	outside
182	195	IMPYVAGGVSYAQV	14	FBCPred	0.5691	0.18	0.95	-0.91	0	5.87	1454.91	outside
206	225	AADDAEIATAQLFDKTKTLV	20	SVMTriP	-0.2272	-0.1	0.04	0.26	-2	4.23	2121.65	outside
10	29	SVIALISASAAQAADVIVPH	20	SVMTriP	0.4093	0.16	1.32	-0.56	-0.5	5.09	1933.52	outside
232	251	GVDFAMTDNVLLRAEYRYSD	20	SVMTriP	0.9109	-0.18	-0.33	0.11	-2	4.23	2335.85	outside
137	156	ANAAFAQGKTSDNVAAVDKH	20	SVMTriP	0.6529	-0.15	-0.42	0.17	0.5	7.09	2015.44	outside
96	115	NNMILGVETDAVWADREDAK	20	SVMTriP	0.4777	-0.19	-0.55	0.41	-3	4.11	2247.75	outside
